# Hyperspectral imaging suggests potential for rapid quantification of fission products in spent nuclear fuel

**DOI:** 10.1038/s41598-025-89338-w

**Published:** 2025-02-13

**Authors:** R. David Dunphy, Andrew J. Parker, Manuel Bandala, Stuart Bennet, Colin Boxall, Patrick Chard, Neil Cockbain, David Eaves, Dave Goddard, Xiandong Ma, C. James Taylor, Richard Wilbraham, Jaime Zabalza, Paul Murray, Malcolm J. Joyce

**Affiliations:** 1 Department of Electronic & Electrical Engineering, University of Strathclyde, Glasgow, G1 1XQ UK; 2 School of Engineering, Lancaster University, Lancaster, LA1 4YR UK; 3 Mirion Technologies Ltd, 207A Cavendish Pl, Risley, Warrington, WA3 6WU UK; 4 United Kingdom National Nuclear Laboratory, Sellafield, Seascale, CA20 1PG UK; 5 Westinghouse Springfields Fuels Ltd, Salwick, Preston, PR4 0XJ UK

**Keywords:** Hyperspectral imaging, Nuclear fission, Uranium dioxide, Waste management, Post-irradiation examination, Electrical and electronic engineering, Techniques and instrumentation, Imaging techniques

## Abstract

An analysis of sintered uranium dioxide has been conducted using a hyperspectral camera sensitive to short-wave infrared wavelengths in the range 949–2472 nm. Three groups of sintered UO_2_ nuclear fuel pellets were prepared and analysed, with stable sub-group surrogates introduced at the preparation stage to emulate the presence of fission product elements. Results show a clear, consistent, and reproducible spectral response across the pellet groups for pure UO_2_. Furthermore, the addition of fission product elements is observed to affect the shortwave infrared response, causing an overall flattening of the spectra. We have shown that this spectral change is correlated significantly with the presence of lanthanides in the fuel matrix. This result could have important potential in post-irradiation examination for quantifying nuclear fuel burn-up and radiotoxicity at discharge, as the hyperspectral imaging setup allows multiple (> 20) samples to be analysed in a single image, captured in under 30 s.

## Introduction

Nuclear reactors generate approximately 10% of electricity produced globally and, with many countries adopting energy production strategies targeting Net Zero and security of supply, this proportion is forecast to increase^1^. Key to the future of nuclear power in this regard is safe, effective and economic reactor operation and compatibility with what are considered effective arrangements for the disposal of spent nuclear fuel (SNF)^2^. To achieve this, it is critical that reactor fuel performance *in-reactor* is understood, and what physical and chemical changes occur to it during and after irradiation. This is achieved currently via a variety of experimental and simulation approaches^3–11^. The chemical and physical characteristics of SNF are relevant to radioactive waste disposal because they provide evidence by which such arrangements can be judged robust^12^, in particular, that they are compliant with post-closure safety cases of geological disposal facilities^13–16^.

One of the primary tools for understanding nuclear fuel performance and the ways in which it changes during in-reactor operation is post-irradiation examination (PIE)^17^. PIE employs a suite of techniques to identify and quantify physical and chemical changes that may have occurred to the fuel during its use, including electron probe microanalysis (EPMA), X-ray powder diffraction, scanning electron microscopy, optical microscopy, neutron radiography, laser ablation inductively-coupled plasma mass spectrometry^7,9–11,17,18^. Of particular interest is measuring and quantifying the presence of fission products in the fuel matrix^9–11,19–22^. In this context the term *fission product* is used as a catchall term to include isotopes and elements that are formed in the uranium as it undergoes nuclear reactions. The term encompasses fragments of the original uranium atom that are direct products of nuclear fission reactions, i.e., isotopes such as ^90^Sr or ^137^Cs; transuranic isotopes produced by neutron capture, e.g., ^239^Pu, ^237^Np, etc.; and ternary fission products, e.g., ^3^H or ^4^He^23^. In addition to isotopic differences, fission products in spent nuclear fuel display different physicochemical interactions with the UO_2_ fuel matrix. Interactions have been observed that form four distinct categories of fission product according to their chemistry^19^: Solid metallic and metallic alloy precipitates (referred to as *ε-particles*)^24^, elements that form oxide precipitates (generally referred to as the *grey phases*)^25^, those that form *solid solutions* that are dissolved and dispersed homogeneously within the UO_2_ matrix^26^, and volatile and inert gases^27^.

The mass of fission products, or the mass of these individual physicochemical groups, within a fuel pellet can provide a direct measure of burn-up, i.e. how much energy was extracted per mass of initial fuel load, as fission products are only formed when the uranium fuel *burns*, i.e. undergoes fission^28,29^. This provides an indicator to the operating conditions within the reactor during use^30,31^. For example, a comparison of the energy produced, measured by the reactor’s thermal output, with how much fuel was burned, through PIE techniques, can show the efficiency of the reactor. Measuring burn-up can also provide granular reactor performance information, such as identifying whether there was uniform burn-up across individual fuel elements and from one element to another by analysing individual fuel pellets from elements located at different locations within the reactor core^32,33^. Therefore, measuring burn-up is of the utmost importance to reactor operators and fuel manufacturers so that they can maximise electricity output per unit of fuel, and to regulators so that they can ensure safety parameters and operating conditions are not compromised. Lastly, the burn-up and presence of radioactive fission products is a fundamental factor that needs to be considered when disposing and storing of SNF^34^.

The principal difficulty in conducting PIE is the intense ionising radiation field that originate from the fission products within SNF^35^. As such, PIE utilises *hot cells* designed to allow the remote handling and manipulation of SNF by operators separated typically by metre-thick borosilicate leaded glass and concrete walls^36^. Furthermore, the techniques employed in PIE, including ASTM standards, often require destructive sample preparation that includes cutting, cross sectioning, embedding in resin, polishing, or dissolution^17,37^. For example, the best-practice technique for measuring the spatial distribution of fission products and the microscale-level burnup within spent nuclear fuel is EPMA, which has a spatial resolution sufficient to identify fission products of a size < 10 μm. This technique requires sample preparation and decontamination within a hot cell before transfer to the EPMA instrument, which itself is heavily shielded to be able to measure the highly radioactive samples^10^. Given the technical complications, there is scope to find and adopt technologies that simplify sample handling and preparation whilst providing detailed and valuable scientific information whilst reducing the risk and cost of such operations.

One potential material identification and characterisation technology that has been applied for a range of purposes, but not PIE, is hyperspectral imaging (HSI)^38–40^. HSI can capture an image across hundreds of contiguous electromagnetic wavelengths, ranging from the visible to near-infrared, and which can be used to distinguish physical and chemical properties of samples under investigation^41,42^. In a pushbroom imaging system such as the one used in this study, a hyperspectral image or *datacube* is constructed using a line-scanning technique where the sensor collects data across a narrow cross-section of the sample^43^, while the sample is moved under the HSI sensor using a motion stage^44–46^. This method captures one line of spatial information at a time, building the image strip-by-strip. In the resulting datacube, each *band* is a greyscale representation of the scene at a narrow range of photon wavelengths, and each *hyperpixel* contains spectral information for a spatial point in the scene or sample^47^.

HSI is similar in principle to Near-infrared (NIR) reflectance spectroscopy, a technique that has been used to identify rare-earth elements (including lanthanides) and uranium in geological contexts^48–50^. While NIR provides spectral averages over a sample, HSI can provide pixel-level resolution of infrared spectral responses^51^. Other advantages of HSI include short acquisition times, typically in the order of seconds to image a sample or area of interest^52^, as well as its non-destructive nature in comparison to methods such as laser-induced breakdown spectroscopy. These factors are attractive in the context of PIE, as they allow simple operation compared to most existing techniques and potential acquisition of data from multiple samples at the same time. This could lead to the rapid identification and quantification of fission products within the nuclear fuel matrix and a measure of the fuel’s burn-up. In this communication, we investigate the use of HSI in the analysis of sintered UO_2_ nuclear fuel pellets and report our initial results on the physico-chemical information the technique could yield.

## Materials and methods

### Nuclear fuel pellet samples

The UO_2_ fuel pellets samples under investigation in this research were divided into three groups based on origin, geometry, and chemical composition. The groups are:

Group A—Pure UO_2_ Advanced Gas-cooled Reactor (AGR) pellets, produced with an annular geometry and cold press and sintered. This group was manufactured by Westinghouse Springfields Fuels Ltd. (Preston, UK) in 2018. Specific detail on the materials is commercially sensitive, however the pellets were manufactured for commercial use *in reactor* and, as such, meet the chemical and physical quality assurance standards associated with products of this nature, i.e., close to stoichiometric.

Group B—Slices of cold press and sintered fuel pellets with a light water reactor (LWR) geometry but a composition consistent with AGR fuels, including both pure UO_2_ pellets and UO_2_ pellets containing non-active fission product surrogates produced at the National Nuclear Laboratory’s Preston Laboratory (Preston, UK). As such, this group is referred to as SIMFuel. A complete description of the preparation and manufacture of this SIMFuel pellet group is given by Hiezl et al*.*^53^ and an in-depth physicochemical investigation, including μRaman analysis, was completed by Wilbraham et al.^54^, highlighting the UO_2_ stoichiometry and the lack of uranic oxidation. For this work, the Group B pellets were classified into two sub-groups, pure UO_2_ (B-1), and doped UO_2_ with non-active fission products and phases expected in simulated advanced gas-cooled reactor fuel with a burn-up of 25 GWd/tU and 100 years cooling time (B-2).

Group C—SPS fuel pellets with an LWR geometry. Like Group B, this group included both pure UO_2_ pellets (C-1) and UO_2_ pellets containing non-active fission product surrogates (C-2 to C-4) and as such is referred to as Split-SIMFuel. For the manufacture of Group C, depleted UO_2_ powder was provided by ABSCO Ltd. (UK). All other chemicals were AnalaR grade or better and supplied by Alfa Aesar (Heysham, Lancashire, UK) or Sigma Aldrich (Gillingham, Dorset, UK). Unless otherwise stated, all chemical weighing, milling steps and SPS were performed under inert atmospheres in positive pressure gloveboxes (N_2_ gas, < 10 ppm H_2_O and O_2_) to restrict the oxidation of the UO_2_ powder.

All samples were kept in storage at room temperature since manufacture and isolated from other sources, samples, and chemicals. Sintered UO_2_ is relatively inert at room temperature and significant oxidation of UO_2_ into other uranic species (e.g., U_3_O_8_) is unlikely in the time since the pellets were manufactured, particularly since Group C were imaged within two weeks of produced. Table 1 provides an overview of the broad physical parameters of pellet groups and sub-groups and the numbering system used to differentiate between them.

**Table 1:** Pellet groups and general information relating to their manufacture, type, and composition.

Pellet Group	ID	Geometry	Subgroup notes/dopant type	Manufacturer	Year of manufacture	Diameter (mm)	Immersion density (g cm^−3^)	Geometric density (g cm^−3^)
A	–	Annular	Pure UO_2_	Westinghouse	2018	14.50 ± 0.01	10.3 ± 0.02	–
B	1	Solid	Pure UO_2_	Hiezl et al*.*^53^	2011	9.39 ± 0.00	10.69 ± 0.04	10.55 ± 0.03
	2		Doped -			9.69 ± 0.00	9.82 ± 0.009	9.69 ± 0.04
C	1	Solid	Pure UO_2_	National Nuclear User Facility—UTGARD	2023	10.42 ± 0.01	–	9.81 ± 0.10
	2		Lanthanide			10.37 ± 0.01	–	9.93 ± 0.10
	3		Grey-phase			10.38 ± 0.01	–	8.90 ± 0.09
	4		ε-particles			10.48 ± 0.01	–	9.59 ± 0.10

### Dopant composition simulation

Group B and C fission product compositions were generated from calculations using FISPIN, the commercial nuclear fuel burn-up code, reported by Hiezl et al.^53^: ^235^U enrichment was set to 2.65 wt%. and an irradiation time of ∼ 4.57 y to give an end-of-life simulated burnup of 25 GWd/tU. 100 years of cooling time was then also assumed. Table 2 shows the calculated blends of oxide precursors for simulated spent fuel (SIMFuel, Group B2) that will lead to fission product dopants with compositions consistent with the FISPIN calculations described in^55^.

**Table 2:** SIMFuel oxide blends Group B2 undoped and doped fuel pellets.

SIMFuel precursor composition	100 years cooling time (wt%)
UO_2_	95.705
Nd2O_3_	0.761
ZrO_2_	0.793
MoO_3_	0.614
RuO_2_	0.512
BaCO_3_	0.328
CeO_2_	0.297
PdO	0.195
Rh_2_O_3_	0.080
La_2_O_3_	0.156
SrO	0.081
Y_2_O_3_	0.095
Cs_2_CO_3_	0.311
TeO_2_	0.073

For Group C, the FISPIN output was separated into the three categories of fission product that have been observed in spent fuel^56^: Metallic precipitates (i.e. ε-particles), those that form oxide precipitates (i.e. *grey phases*) and those that form solid solutions within the continuous UO_2_ matrix. Note: due their relative sintering difficulty, the fourth category of fission product (inert fission gases/volatiles) does not form part of this study. The high radioactivity isotopes were replaced with lower activity surrogates; due to their miscibility in urania and close periodicity, Pu, Am, Cm, Sm, Pr, Pm and Np were represented by additional U to generate SIMFuel compositional mixes for each sub-group, so called *Split*-SIMFuel^57^. Table 3 shows calculated blends of oxide precursors for each sub-group that, upon blending and sintering, will lead to fission product dopants with compositions consistent with the FISPIN calculations described in^53,55^.

**Table 3:** Split-SIMFuel oxide blends for each dopant category.

Split-SIMFuel precursor composition	100 years cooling time (wt%)
Group C-2, Total Solid Solution wt.% = 0.813%	
UO_2_	99.187
Nd2O_3_	0.483
CeO_2_	0.193
La_2_O_3_	0.096
Y_2_O_3_	0.041
Group C-3, Total Grey Phase wt% = 0.413%	
Oxides:	25 GWd/t U (Low-doped)
UO_2_	99.587
BaZrO_3_	0.343
SrZrO_3_	0.07
Group C-4, Total ε Phase wt% = 0.719%	
UO_2_	99.281
MoO_3_	0.334
RuO_2_	0.257
PdO	0.090
Rh_2_O_3_	0.038

### Ball milling and SPS of split-SIMFuel group C pellets

Based on the data of Table 2, 40 g blends were prepared for each Split-SIMFuel dopant sub-group. Stainless Steel 120 ml volume milling jars and 10 mm milling media were used. Each material blend was milled in a planetary ball mill (PM100, Retsch) for 20 min at a speed of 120 rpm.

Sintering was performed using a SPS630-sx system (Suga Co., Ltd., Japan). Approximately 7 g of the milled powders was loaded into graphite dies of diameter 10.5 mm. The inner die surface was covered by a 0.25 mm layer of Grafoil® to prevent reaction of the Split-SIMFuel pellet with the die wall and uptake thermal expansion. 10 mm cylindrical graphite punches were inserted into both ends of the die. The surface of each punch in contact with the powder was covered with a further 10 mm piece of Grafoil® for ease of release of the Split-SIMFuel pellet from the die after sintering. The die was wrapped with graphite felt, and graphite felt was also used at the top and bottom of the die to reduce the heat loss from the graphite surface and decrease the thermal gradient across the die body/punch ends.

The die assembly was placed in the SPS sintering chamber via an incorporated positive pressure glovebox and the chamber was depressurized to < 10 Pa. Before heating and throughout sintering a constant pressure of 40 MPa was applied. Temperature was measured by a pyrometer with a lower limit of detection of 300 °C. Thus, the programmed heating schedule was only initiated after the temperature of the die surface (measured through a ~ 10 mm opening cut in the felt) reached a stable ramp speed between 300 and 600 °C (typical heat up over this range 100 to 150 °C a minute). Samples were sintered from 600 to 1000 °C at a rate of 60 °C a minute, before slowing to a rate of 25 °C a minute from 1000 to 1050 °C. The maximum temperature of 1050 °C was held for 5 min before release of the applied pressure and removal of all current at run end.

After removal from the graphite die, surface Grafoil® was ground off upper and lower Split-SIMFuel pellet faces using 600 and 1200 grit SiC paper, leaving final pellets with approximate dimensions of 10 mm diameter × 8 mm thick and densities 83 to 92% of the theoretical density.

### Hyperspectral imaging equipment

**Fig. 1:**
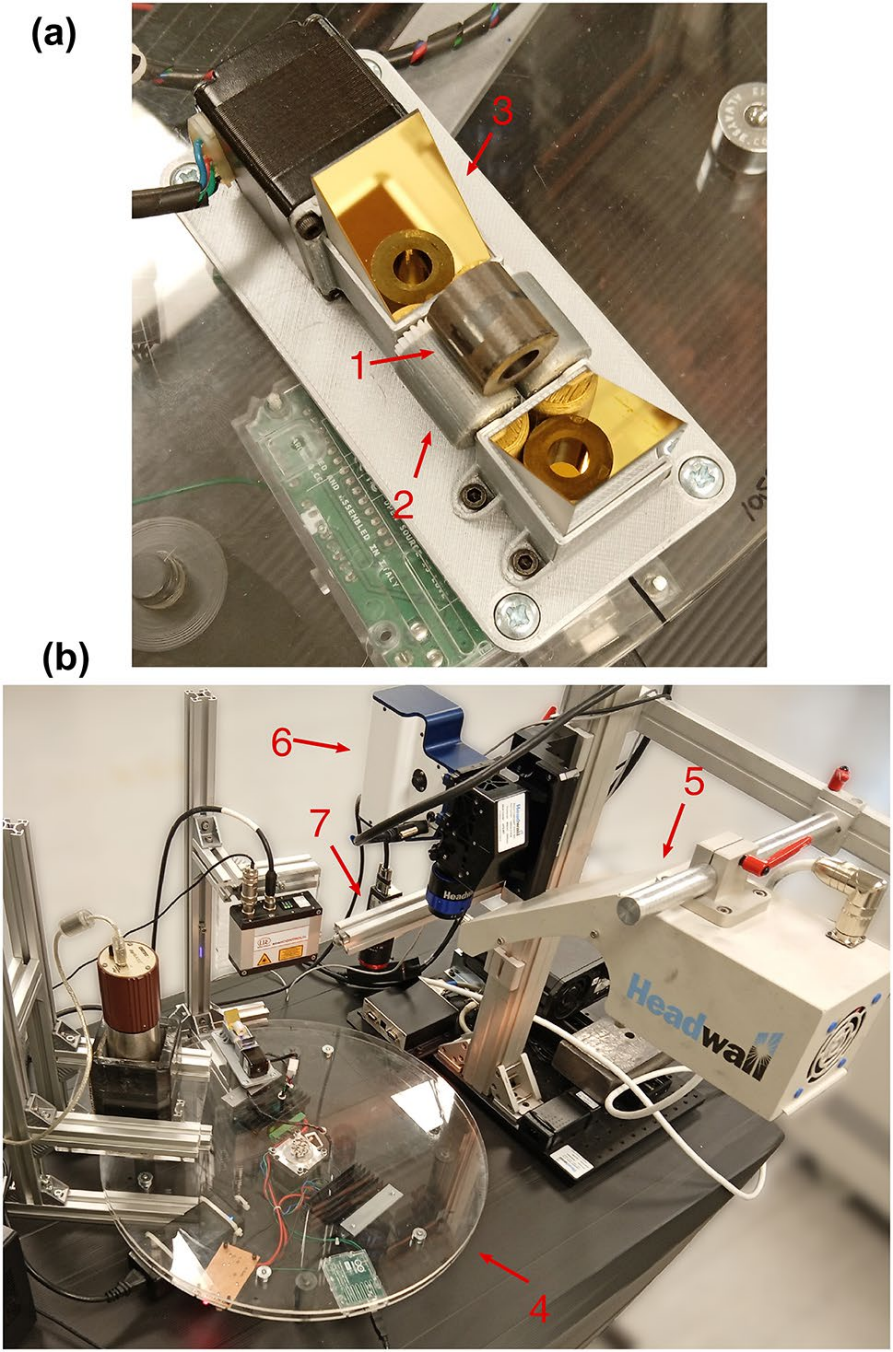
(**a**) Photograph of a Group A pellet placed on the pellet stage, (**b**) Photograph of the hyperspectral imaging apparatus, (1) annular pellet, (2) motorised mechanical rollers, (3) Gold-plated prism mirrors angled at 45°, (4) Rotating turntable, (5) quartz-tungsten-halogen light source, (6) Headwall SWIR-640 camera, (7) RGB camera.

**Fig. 2:**
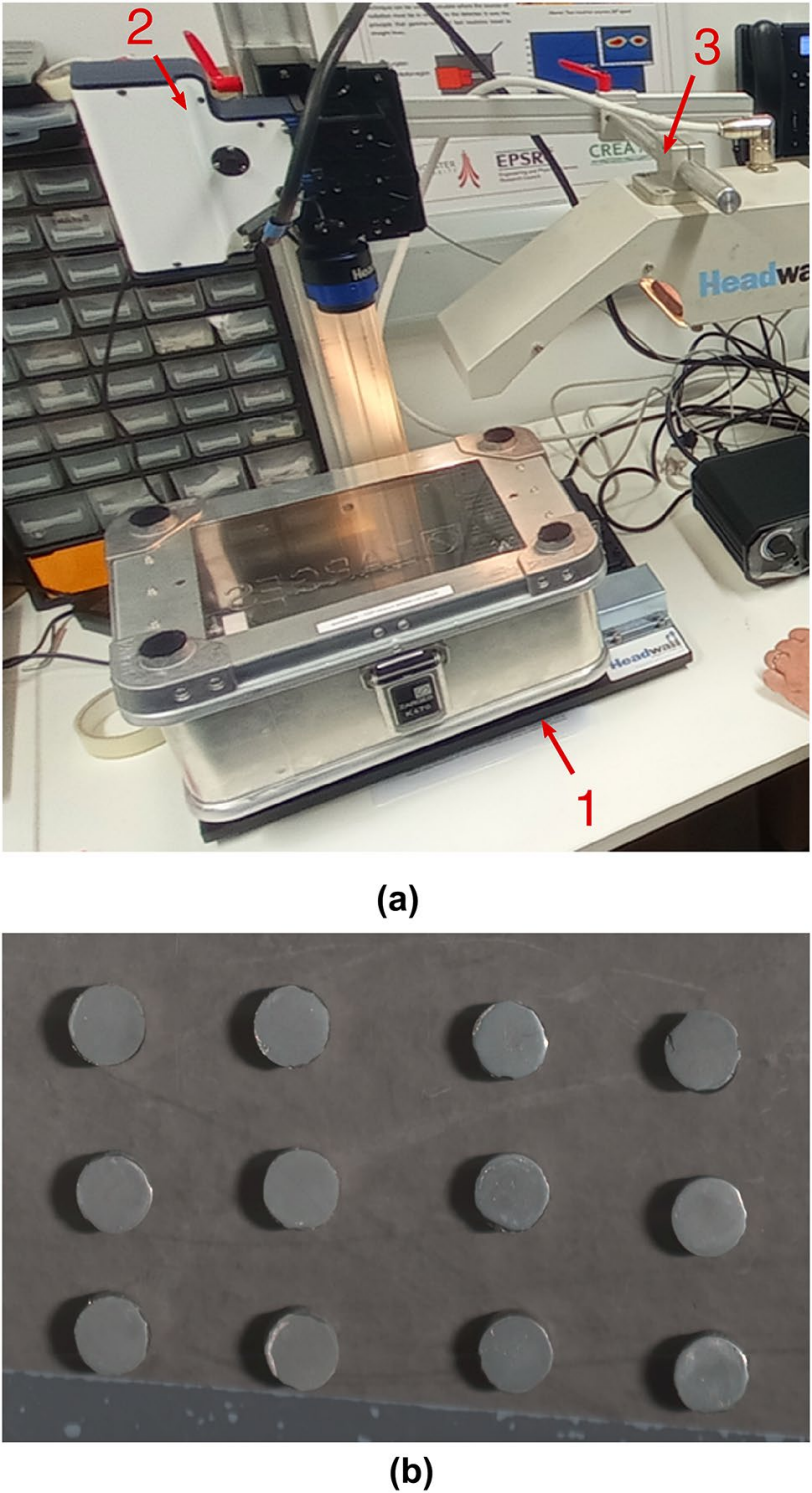
(**a**) Photograph of the hyperspectral imaging setup used for the SIMFuel (Group B) and Split-SIMFuel (Group C) pellets. A box containing pellets with the UVFA7980 glass-top lid (1) is placed on the translation stage beneath the SWIR hyperspectral camera (2). The cross-section of the sample is illuminated using a halogen lamp (3). (**b**) Top-down view of the contents of the box, Split-SIMFuel pellets. The pellets are placed in a grid on a dark background to reduce light scattering.

The pellet groups were imaged using a pushbroom hyperspectral imaging system, consisting of a Headwall Micro-Hyperspec Short-wave Infrared (SWIR) 640 imager with a 25 mm lens at F2.5, a 225 mm linear translation stage, and a quartz-tungsten-halogen light source. The image captures frames with a horizontal resolution of 640 pixels, and a wavelength resolution of 272 bands in the range 889 nm to 2508 nm. For the Group A pellets, roller bearings were used to rotate the pellets in place of the translation stage, allowing the side faces of the pellets to be imaged, as shown in Fig. 1. For this experimental configuration, no container was required. Due to laboratory restrictions, Group B and C pellets were imaged in an airtight Zarges K470 container through a UVFA7980 fused silica glass lid, as shown in Fig. 2a. Both end surfaces of each pellet were imaged from above with the exception of one SPS lanthanide pellet, which was imaged from the side due to damage. Figure 2b shows an RGB image of 15 Split-SIMFuel Group C pellets arranged within the Zarges K470 container. To reduce light scattering, the Group B and C pellets were placed atop a dark background.

All images were one-point calibrated to a Spectralon® reference tile, a material known for its high reflectivity and consistent performance across a wide range of wavelengths. Calibrating to a known reference normalises the data against a standard baseline, adjusting the spectra for biases caused by the spectral response of the illumination source and the quantum efficiency of the sensor. Bands 1 to 10 and 267 to 272 were discarded due to high levels of sensor noise, leaving 256 bands sensitive to SWIR wavelengths in the range 949 to 2472 nm.

### Spectral analysis

**Fig. 3:**
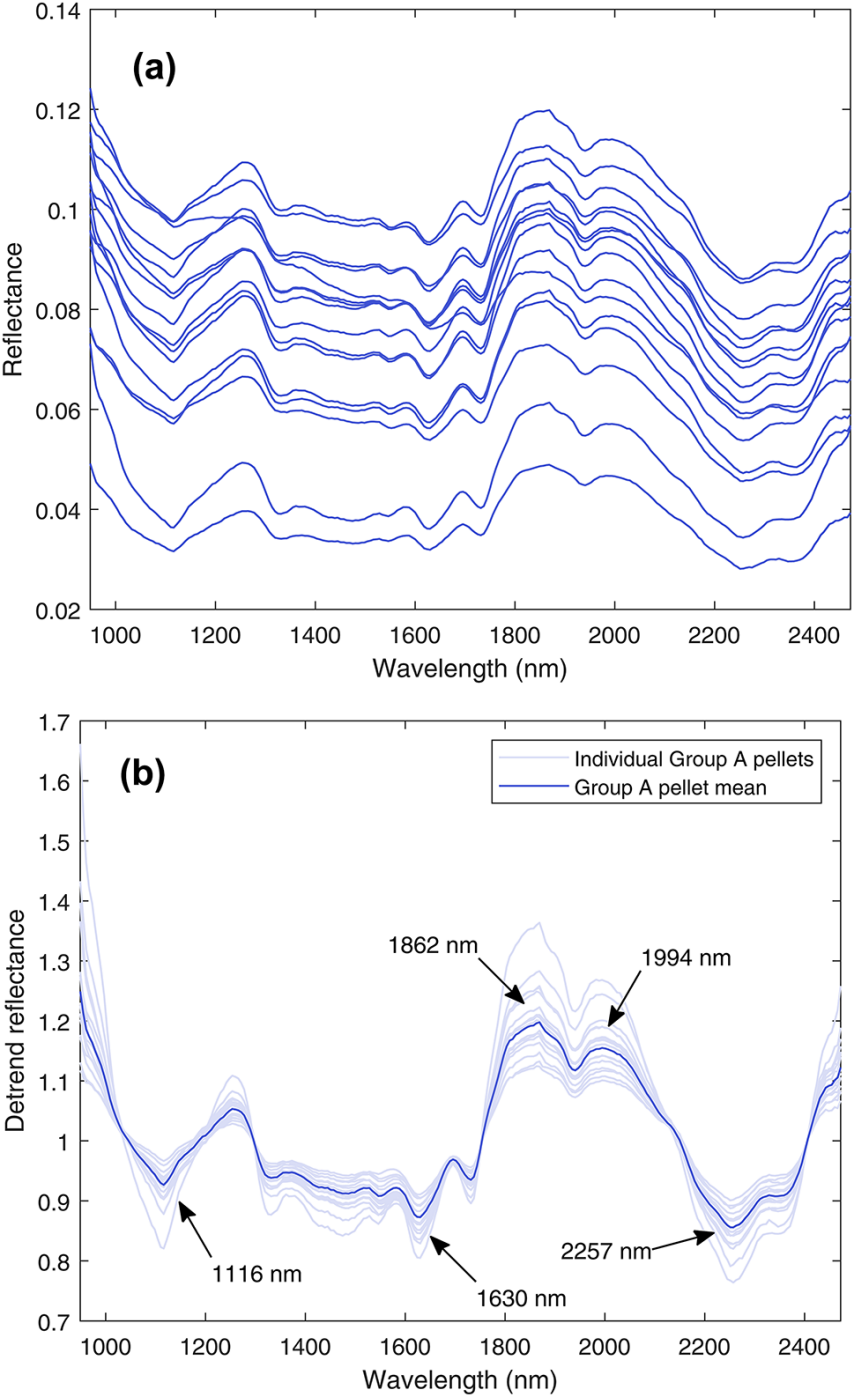
SWIR reflectance spectra for the 16 pure UO_2_ pellets in Group A (**a**) before and (**b**) after detrending. The spectrum is characterised by consistent absorbance bands at 1116 nm, 1630 nm, and 2257 nm as well as two distinctive spectral peaks at 1862 nm and 1994 nm.

Regions of interest (ROIs) were created by manually selecting a set (or *mask*) of pixels constituting the surface area of the pellets. To remove areas of specular reflection that distort the spectral response, hyperpixels with a mean reflectance value greater than an empirically determined threshold value of 0.4 were removed from the mask. The 256-value reflectance spectra reported for the pellets are the mean of the hyperpixels in the respective ROIs. Figure 3 shows the reflectance spectra for the pellets in Group A. The spectral features present in the samples were found to be low in amplitude compared to the overall trend of the data, and some spectra were further dominated by linear trends resulting from scattering that is highly sensitive to the incident angle of the light. Intraclass variance was reduced by normalising the signals using quadratic detrending^58^. Detrending of a reflectance spectrum $R$ is applied to the absorbance $A=-{\text{log}}_{10}R$ by subtracting the quadratic fit of the absorbance ${Q}_{A}$, resulting in detrended reflectance

$$\widetilde{R}={10}^{{Q}_{A}+{\text{log}}_{10}R} .$$

This method was found to reduce intra-class variance more than other widely used normalisation methods such as standard normal variate.

## Results

**Fig. 4:**
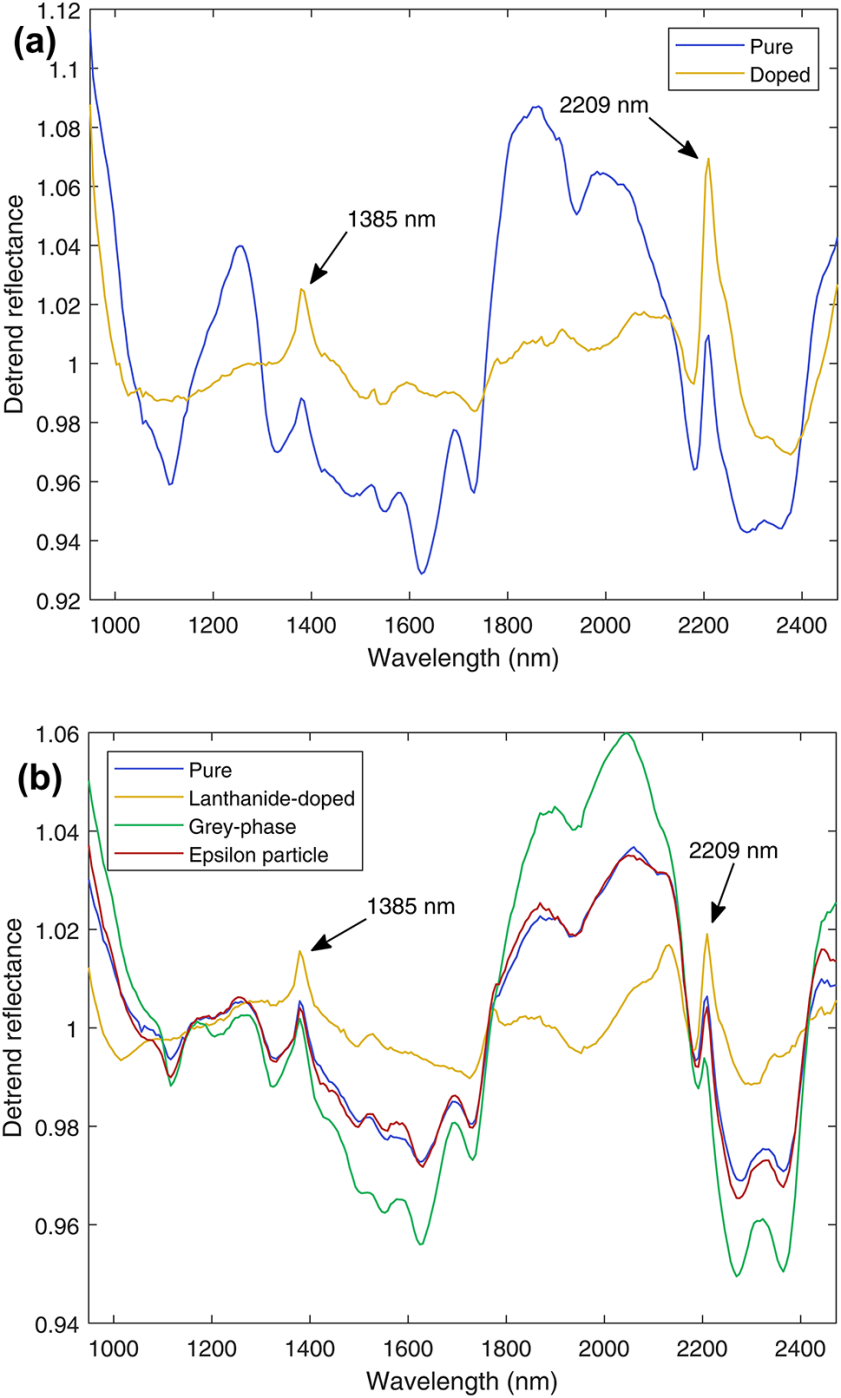
Mean detrended SWIR spectra for (**a**) pure and doped SIMFuel pellets (Group B), and (**b**) Split-SIMFuel (Group C). Two narrow spectral peaks at 1385 nm and 2209 nm present in all spectra are artefacts caused by absorbance bands in the UFVA7980 glass lid through which the pellets were imaged. With the exception of these, the doped Group B-2 pellets and lanthanide-doped Group C-2 pellets have significantly flatter responses than undoped pellets, with the features identified in Fig. 3 suppressed relative to other pellet classes.

Figure 3 shows the mean detrended shortwave infrared (SWIR) spectrum for each of the pure UO_2_ annular pellets imaged (Group A). Several spectral features are observed that are consistent across all Group A pellets, including an absorbance band at 1116 nm, a series of five small absorbance features between 1300 and 1750 nm, the most prominent of which is at 1630 nm, and a pair of absorbance bands at 2257 nm and 2359 nm. The spectra are dominated by a pair of spectral peaks at 1862 nm and 1994 nm. These features are also observed in the spectra for the pure UO_2_ pellets of the SIMFuel Group B-1 (Fig. 4a) and the Split-SIMFuel Group C-1 (Fig. 4b). This consistent response suggests these spectral features are typical of sintered UO_2_ independent of sintering technique used, i.e., conventional cold press and sinter or spark plasma sintered (SPS). It should be noted that the narrow spectral peaks at 2209 nm and 1385 nm, which are present across all pellet types in Groups B and C, but not the spectra of Group A, are calibration artefacts introduced by the glass window in the Zarges K470 container.

The distinctive, adjacent spectral peaks at 1862 nm and 1994 nm are not present in the spectra collected for the doped SIMFuel Group B-2 pellets in Fig. 4a. Furthermore, all other absorbance features present in the pure UO_2_ are weaker in this pellet group, leading to a flatter overall response across the measured SWIR wavelengths. This suggests that accumulated fission products (or simulated fission products) within the fuel matrix change the material’s SWIR reflectance characteristics sufficiently to the point where they may be differentiated from the SWIR response of pure UO_2_ pellets using HSI.

From Table 2 it can be seen that there are a large number of elements appearing as fission products in UO_2_ fuel with a simulated burn-up of 25 GWd/tU. These elements are diverse in terms of their chemistry and vary in the manner of their interaction with the UO_2_ fuel matrix, and the resultant microstructure of the SIMFuel Group B-2 pellets contain lanthanides in solid solution with the UO_2_ matrix, and grey phases and metallic precipitates. As such, attributing the change in SWIR response between the pure and the doped pellets to one or more of these simulated fission categories from the data in Fig. 4a alone is problematic. However, the Split-SIMFuel Group C pellets allow a more detailed insight as to the causes of this spectral change, as the fission products have been grouped together according to the general chemistry of fission products observed in spent nuclear fuel, i.e., lanthanide, grey phase, or ε-particles, such that the pellet sub-groups contain only one fission product type, shown in Table 3, unlike SIMFuel Group B-2.

A comparison of the Split-SIMFuel Group C SWIR spectra shown in Fig. 4b shows that the lanthanide-containing pellets sub-group (Group C-2) has a flat response, with the main absorbance features from the pure UO_2_ spectra suppressed. The other two dopant-containing sub-groups, grey phase and ε particles, show a SWIR response closer to that of pure UO_2_. Interestingly, the presence of grey-phase (Group C-3) fission products appears to enhance the dominant 1862 nm and 1994 nm peaks relative to pure UO_2_.

Comparing the SWIR response of the SIMFuel Group B-2 (Fig. 4a) and Split-SIMFuel Group C pellets (Fig. 4b), it is apparent that the Group B-2 spectra are consistent with the response from Group C-2, the lanthanide sub-group. This is despite the Group B-2 pellets containing dopants across lanthanide, grey-phase, and ε particle sub-groups. Indeed, it appears that the presence of lanthanides in the UO_2_ matrix overrides the grey-phase enhancements to the dominant 1862 nm and 1994 nm features.

**Fig. 5:**
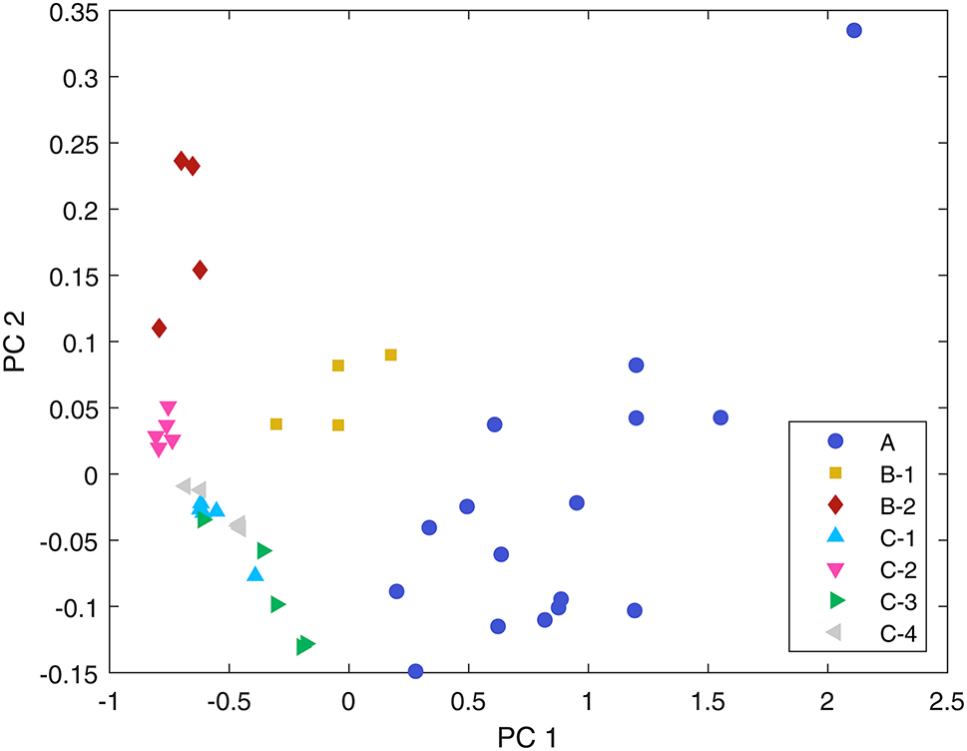
The first two principal components of each mean pellet spectrum. Clear clusters between pellet groups can be observed, with considerable overlap between Group C pellets not doped with lanthanides. The lanthanide-doped Split-SIMFuel Group C pellets (C-2) and doped SimFuel pellets (B-2) form a cluster with similar values in the first principal component.

The relative similarity of lanthanide-containing pellets is further demonstrated by principal component analysis (PCA). Figure 5 shows a scatter plot of the first two principal components for each detrended pellet spectrum. Lanthanide containing pellets (Groups B-2 and C-2) form a cluster on the left of the plot, while there is considerable overlap between non-lanthanide containing pellets from Split-SIMFuel Group C. The clear separation between Group A pellets and other pure pellets can be attributed to the calibration artefacts that are present across all other pellet groups.

Based on the observations across the pellet spectra, an index, $Z$, is proposed to quantify differences by comparing the magnitude of the spectral peak at 1870 nm with the peak absorbance at 2257 nm, normalised to the range of the detrended data:

$$Z=\frac{{\widetilde{R}}_{1870 {\text{nm}}}-{\widetilde{R}}_{2257 {\text{nm}}}}{\text{max}\left(\widetilde{R}\right)-\text{min}\left(\widetilde{R}\right)}$$

Using this index results in a $Z$-score ranging between -1 and 1 indicative of the prominence of the spectral feature observed in Fig. 3, where a score of 1 indicates that the difference between detrended reflectance values at 1870 nm and 2257 nm (${\widetilde{R}}_{1870 {\text{nm}}}$ and ${\widetilde{R}}_{2257 {\text{nm}}}$, respectively) dominate the spectrum, a score of 0 indicates the absence of a spectral feature in this range, and a score of − 1 indicates that the spectrum is dominated by an inverse spectral feature (i.e., a relatively higher value at 2257 nm caused by an absorbance feature around 1870 nm).

**Fig. 6:**
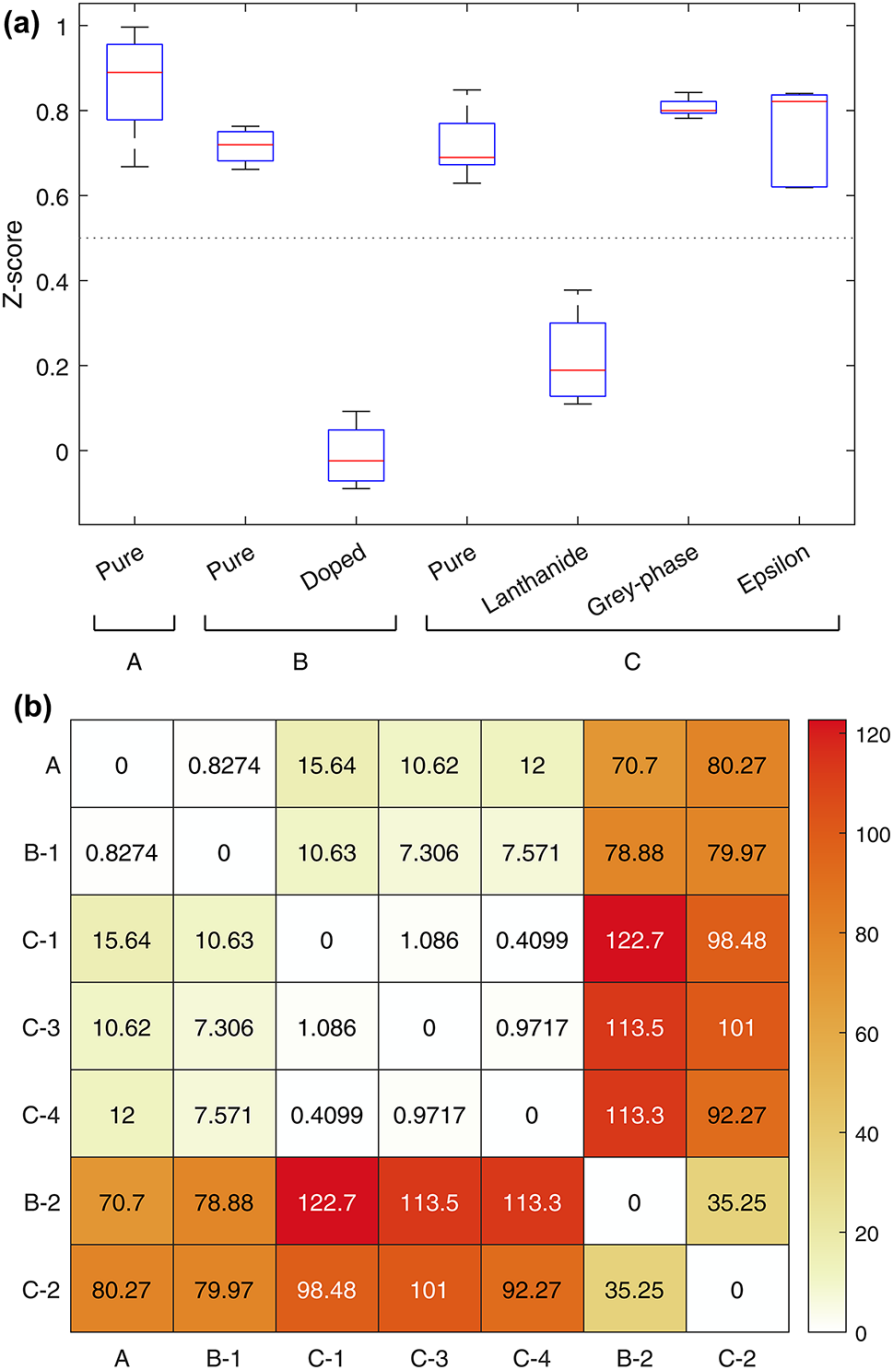
(**a**) Box plot showing the index of the two main features at 1870 nm and 2257 nm. The extent of the box covers the 25–75th percentiles, with the red line indicating the median value. The horizontal line at Z = 0.5 marks the separation between the lanthanide-containing pellet spectra and all other pellet spectra. (**b**) Pairwise spectral information divergence (SID) scores for each of the pellet groups. SID is calculated based on bands 1 to 205 (949–2167 nm) in order to avoid the glass lid artefact around 2209 nm. Lower scores indicate greater spectral similarity. As with the Z-score, this measure shows that doped SIMFuel (Group B-2) and lanthanide-doped Split-SIMFuel (Group C-2) pellets are most similar to each other, and different from other pellet groups.

Figure 6a shows a box plot of the index values for the mean pellet spectra. It is evident that there is no significant difference between the pure UO_2_ (Groups A, B-1, C-1), grey phase(Group C-3), and ε particle (Group C-4) sub-groups from the image data captured as there is significant overlap between the $Z$ values for these categories, with values consistently above 0.6. In contrast, the doped SIMFuel (Group B-2) and lanthanide-doped Split-SIMFuel (Group C-2) sub-groups exhibit low scores, with a median score of − 0.02 for Group B-2 and 0.19 for Group C-2. The statistical relationship between pure and fission product-doped sub-groups was tested using a Welch’s t-test. Comparing the SIMFuel B-2 sub-group and the pure A group pellets, there is apparent statistical independence between the two groups, with a *p*-value of $5.4\times {10}^{-6}$. Likewise, the Split-SIMFuel C-2 sub-group is statistically independent from the pure pellets (Group A) with a *p*-value of $3.5\times {10}^{-5}$. This indicates a real difference between the SWIR spectral response detected from these groups.

Spectral similarity can also be measured using spectral information divergence (SID) which treats the spectral signatures of materials as probability distributions and estimates the difference between spectra by calculating the divergence of these distributions, with low scores indicating greater spectral similarity^59^. Figure 6b shows the pairwise spectral similarities for the sub-groups using SID. Like the $Z$-score, this metric also shows that the pure pellet classes are more similar to each other than to the doped or lanthanide-doped pellets, while the doped and lanthanide-doped pellets are most similar to each other. The SID scores shown are for the spectral range from 949 to 2167 nm (bands 1–205). This range was chosen to omit the false feature at 2209 nm caused by the transmittance of the glass lid for the SIMFuel Group B and Split-SIMFuel Group C pellets. Including the entire SWIR spectral range resulted in higher SID scores between the doped and lanthanide-doped spectra, as the flatter overall spectral signal makes these classes more sensitive to the calibration artefacts. As this range does not cover the absorbance band at 2256 nm, the spectral similarities between the pure pellet groups are caused by the secondary absorbance features, such as those at 1116 nm and 1630 nm. Table 4 shows the measured pellet densities and Z-scores used to quantify the spectral changes observed and indicates no correlation between pellet density and spectral change.

**Table 4:** The measured densities of the pellets and the calculated, mean Z-score taken from the SWIR spectra.

Pellet group	ID	Immersion density (g cm^−3^)	Geometric density (g cm^−3^)	Mean Z-score
A	–	10.3 ± 0.02	–	0.87 ± 0.10
B	1	10.69 ± 0.04	10.55 ± 0.03	0.72 ± 0.04
	2	9.82 ± 0.009	9.69 ± 0.04	− 0.01 ± 0.08
C	1	–	9.81 ± 0.10	0.72 ± 0.08
	2	–	9.93 ± 0.10	0.22 ± 0.11
	3	–	8.90 ± 0.09	0.81 0.02
	4	–	9.59 ± 0.10	0.75 ± 0.12

The combination of these analytical methods shown in Fig. 6a and b serves to further highlight divergence in spectral response when the lanthanide materials are introduced into the UO_2_ matrix. This represents the core finding of this work, that the presence of lanthanides in a nuclear fuel pellet can be identified rapidly using hyperspectral imaging.

**Fig. 7:**
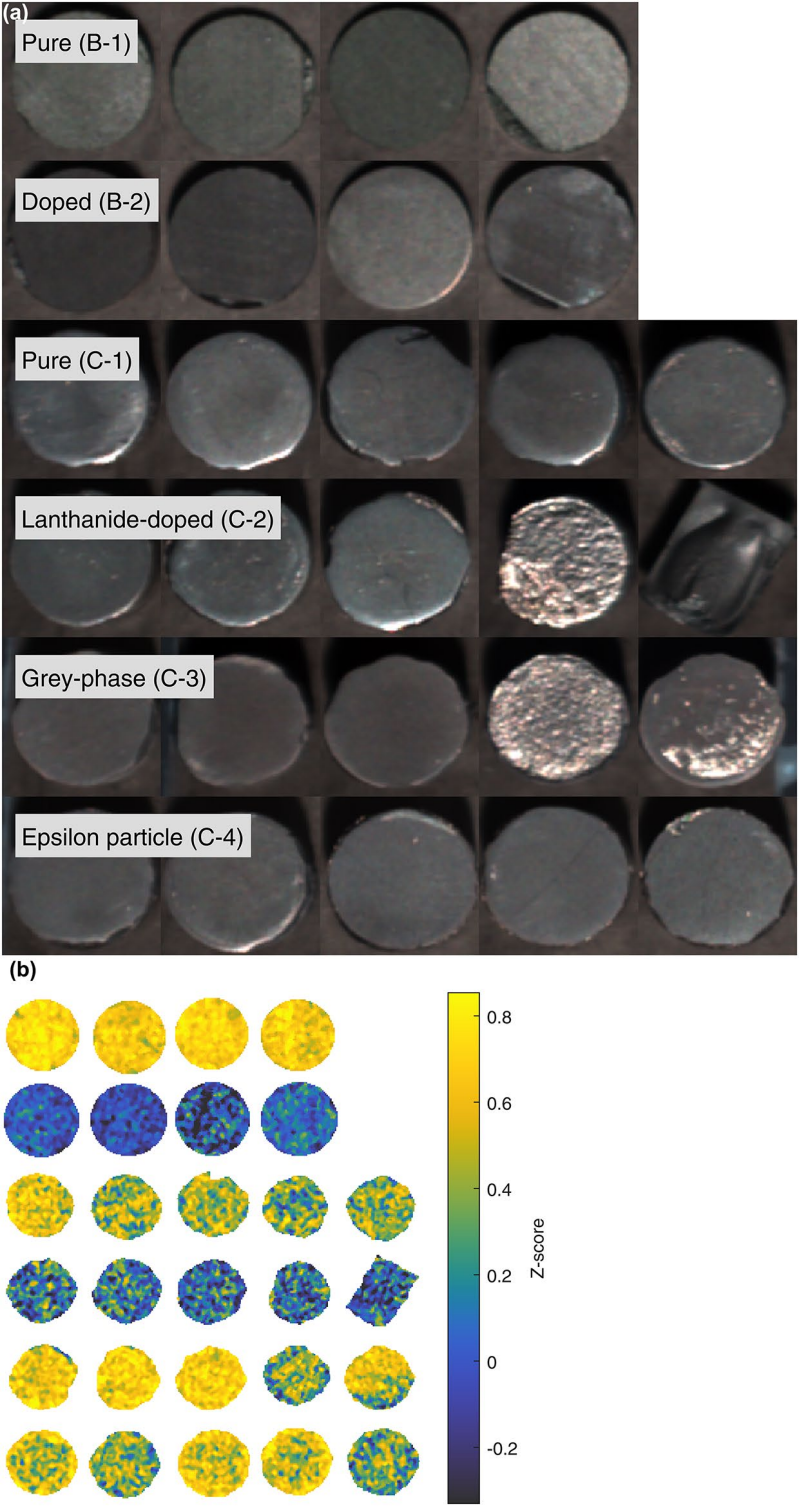
Top views of Group B and Group C pellets. (**a**) False-colour image (bands 109, 42, and 15 of the SWIR hyperspectral data) of pellet surfaces. (**b**) Heatmaps showing the Z-score for each hyperpixel in the ROIs. Doped pellets (B-2) and lanthanide-doped pellets (C-2) have lower scores than other pellet groups.

Furthermore, a major advantage of hyperspectral imaging over conventional spectroscopy techniques such as NIR spectroscopy is the acquisition of spatial data in addition to the spectral dimension. By treating each hyperpixel as a separate spectrum, it is possible to discover spatial distributions that are not apparent from single-point spectra. Figure 7a shows false-colour representations of SIMFuel and Split-SIMFuel pellet groups generated from bands 109, 42, and 15 (1594 nm, 1194 nm, and 1033 nm, respectively) of the hyperspectral datacube. Figure 7b shows a heatmap of $Z$-scores for the SIMFuel Group B and Split-SIMFuel Group C pellet surfaces presented in Fig. 7a, which demonstrates that while the trends observed in the mean pellet spectra are borne out in the distribution of scores, there is some spatial variation for some pellet groups. In particular, the lanthanide-doped Split-SIMFuel Group C-2 pellets exhibit small *hot spots* where the $Z$-scores are higher than the surrounding areas. These hot spots are the cause of the higher average scores relative to SIMFuel Group B-2 seen in Fig. 7a, and may be an artefact of the differing manufacturing methods employed between SIMFuel Group B and Split-SIMFuel Group C, respectively.

## Discussion

The most direct comparison to our study is the work of Klunder et al*.*^60^ who used NIR absorbance spectroscopy to analyse uranic compounds, including UO_2_ powder. However, given important differences in sample preparation and imaging protocol, a comparison is inappropriate. Other works in this area are mostly directed towards uranic mineral materials from geological exploration literature, e.g., coffinite (U content ≥ 72%), which give rise to complex spectra resulting from a multi-elemental composition. Nevertheless, there are apparent consistencies between the NIR spectral responses and the SWIR HSI data reported here. Gajek et al.^61^ and Turner^50^ both identify significant absorption features in the 1114–1116 nm and 1630–1650 nm wavelength ranges in UO_2_ and uranic minerals, respectively, which are consistent with absorption features found in our spectra. Hebert et al*.*^62^ were able to quantify the abundance of coffinite within a mixed-mineral sample from the magnitude of the 1135 nm absorption band, which is not readily distinguishable in our spectra, which has also been reported as a weak absorption feature for U^4+^ [Turner]. But, from the available literature it is evident that prominent uranium SWIR absorbance features appear around 1116 nm and 1630 nm that are attributed to U^5+^ and U^4+^_,_ respectively^50,61^. Other consistencies included, but limited to, the 1912 nm band indicative of H_2_O content in the sample^63^. Given all pellets used in this study are sintered, either cold press or by SPS, the moisture content will be negligible.

It is unclear why introducing low percentages of lanthanides into a homogenous uranium dioxide material, that may or may not contain small quantities of other fission products, has a flattening effect across the measured SWIR wavelengths. In contrast, Turner^50^ identified the presence of lanthanide species, including neodymium and lanthanum, in zircon (a mineral with high uranium content). Unlike this work where SIMFuel Groups B-2 and Split-SIMFuel Group C-2 have no dominant absorbance band between 1116 and 1630 nm, the spectral response and absorbance features attributed to uranium in the minerals were not diminished or flattened in this range. Any of the potential features in the 1350–1650 nm range which may be attributed to uranium are also significantly altered. While zircon is a different class of material, the reason for the spectral response changes is unclear, and understanding this will be the subject of future studies.

The absence of any adsorption features that could be attributed to either grey phase or ε particles is likely due to the resolution of the SWIR camera setup adopted. These fission product types appear as precipitates within the UO_2_ structure with reported diameters typically between 2 and 35 μm^24,25^. This is far smaller than the resolution of the SWIR 640 imager (approximately 0.8 × 0.8 mm). With refinement to the experimental setup and adoption of magnifying lenses, these particles may become detectable.

Nonetheless, the SWIR response is clear, consistent, and repeatable across the pellet groups and subgroups studied in this work, and consistent with similar works in literature. The combined spectral and spatial data, collected using hyperspectral imaging such as the setup employed here, allows multiple (> 20) nuclear fuel pellets to be analysed in a single image and captured in under 30 s. The data generated from these images has shown statistically significant SWIR spectral differences correlated to the presence of lanthanides in the fuel compared to the SWIR response of sintered uranium dioxide and that this consistency extends to the sintering technique employed and does not appear to be related other physical parameters, e.g. sample density. It is also evidence that negligible oxidation of the UO_2_ has occurred in the pellets, given the different manufacturing methods and ages between the pellet groups, as the spectra are consistent. The results also show that HSI could provide spatial information on the location of lanthanides fission products within an individual pellet, as shown in Fig. 7, at a resolution on the millimetre scale. Locating specific areas of fission product accumulation in a fuel pellet increases the granularity in measuring fuel burn-up uniformity from the fuel element level to the sub-pellet level, as provided by EPMA. Despite an order of magnitude difference to EPMA^9–11^, the finding is significant as further experimental and technology optimisation could significantly improve the HSI systems resolution. This could be used in the detection of non-uniform burn-up over a single pellet, providing forensic-level information on reactor performance.

The rapid identification, quantification, and location of fission products such as lanthanides within the fuel matrix has the potential to provide an assessment of quantifying nuclear fuel burn-up. Measuring fuel burn-up provides information on the *in-reactor* performance of the nuclear fuel and the reactor conditions which is valuable to energy generators, fuel manufacturers, and government regulators across a range of economic, safety, and political contexts. In addition, fuel burn-up data is also used to inform decision making processes regarding the future long-term storage requirements of spent fuels. Finally, there may be further application for quality assurance purposes in the manufacturing of nuclear fuels that include lanthanide-group burnable poisons, e.g. gadolinium.

To understand the physicochemical mechanisms for these spectral changes further investigation and analysis is ongoing, including μRaman spectroscopy and scanning electron microscopy of the pellet Groups. Furthermore, experiments are in preparation that will attempt to quantify lanthanide content in uranium dioxide pellets, based on the spectral changes resulting from its presence. Lastly, work is ongoing to understand the use, performance, and robustness of hyperspectral cameras in high radiation environments via standoff means such as longer focal lengths or fibre optic cables.

## Data Availability

The datasets generated and/or analysed during the study are not publicly available due to commercial interests from project partners Westinghouse Springfields Fuels Ltd and the National Nuclear Laboratory, whose samples were used to generate the data. However, the datasets may be available from the corresponding author on reasonable request pending agreement with all project collaborator organisations.
